# Distribution and characterization of extrachromosomal circular DNA in colorectal cancer

**DOI:** 10.1186/s43556-022-00104-0

**Published:** 2022-12-02

**Authors:** Zhehang Chen, Yadong Qi, Jiamin He, Chaochao Xu, Qiwei Ge, Wei Zhuo, Jianmin Si, Shujie Chen

**Affiliations:** 1 Department of Gastroenterology, Sir Run Run Shaw Hospital, Zhejiang University, Hangzhou, Zhejiang, Province China; 2 Institute of Gastroenterology, Zhejiang University, Hangzhou, China; 3 Department of Gastroenterology, Second Affiliated Hospital of Zhejiang University School of Medicine, Hangzhou, Zhejiang, Province China; 4 Cancer Center, Zhejiang University, Hangzhou, China; 5 Department of Cell Biology and Department of Gastroenterology, Sir Run Run Shaw Hospital, Zhejiang University School of Medicine, Hangzhou, China

**Keywords:** Extrachromosomal circular DNA, Circle-seq, Bioinformatic anaylsis, Colorectal cancer, Oncogene amplification, Differential expression

## Abstract

Extrachromosomal circular DNA (eccDNA) has been shown to play an important role in the amplification of tumor genes and the maintenance of intra-tumor genetic heterogeneity, although its complex functional mechanism still remains to be elucidated. As the top three common malignancies in the world, colorectal cancer (CRC) has been threatening human life and health, whose tumorigenesis and development may have elusive connection with eccDNAs. Here, we described the extensive distribution of eccDNAs in the CRC tissues using Circle-seq, which range in size from hundreds to thousands of base pairs (bp). The distribution in tumor tissues had aggregation and tendency compared with random in tumor-adjacent tissues, accompanied with smaller and more regular circle lengths. After sequencing and restoring, we found that the shedding sites of eccDNAs in CRC had similar tendency in chromosome distribution, and focused on tumor-associated genes. Meanwhile, we combined RNA sequencing to explore the correlation of eccDNA differential expression in the gene transcription and signaling pathways, confirming a connection between eccDNA and RNA somewhere. Subsequently, we validated eccDNAs in CRC cell lines and the potential consistency of the junction sites of eccDNAs in CRC tissues and cell lines. Using fragments of the cationic amino acid transporter SLC7A1 to synthesize eccDNAs, we discovered the role of eccDNAs in different regions within the gene.

**Supplementary Information:**

The online version contains supplementary material available at 10.1186/s43556-022-00104-0.

## Introduction

EccDNA is isolated from the chromosomes and circularized, widely presenting in both normal and cancerous tissues of eukaryotic organisms, where it can remain in the nucleus or be free in the plasma [1–3]. The size of eccDNA ranges from tens to millions of base pairs, some carry only promoter or exon elements [4], while others contain complete genes [5, 6], whose mechanism of generation and function is different and intricate [2, 7, 8]. EccDNA has been reported to involve in the amplification of oncogenes and the maintenance of intra-tumor genetic heterogeneity [2, 9], and it may play an important role in tumor surveillance, early diagnosis, treatment, and prediction of cancer.

Despite being one of the most common cancers in the world, the association between CRC and eccDNA remains unexplained [10]. More attention has paid to the relationship between CRC and double-minutes (DMs), whose formation was still controversial but certainly a type of circular DNAs [10, 11]. So by the same token, the distribution and effect of eccDNAs in CRC remain elusive. EccDNA is a complex and multi-step product, which is shed from chromosomes and cyclized [2, 8]; Some carry gene segments on different chromosomes that can be rearranged and combined to perform complex functions [2, 6, 12].

By using different sequencing methods, the eccDNAs are broken into fragments of different sizes and then mapped onto the chromosome to restore the corresponding genes and shedding sites [3, 13]. Accordingly, we carried out descriptive study of eccDNA in CRC, and found that the length distribution and chromosome distribution of eccDNA were aggregated in CRC. However, there are technical predicaments in the functional exploration of eccDNA at present, and few experiment-related articles have been reported [8, 14]. Because of random shedding, it is difficult to explore the function of specific circular DNA [15]; the lack of replicating elements makes it hard to exist in cells for a long time like plasmids [16]. Instead, eccDNA can be synthesized, mainly by the ligase-assisted minicircle accumulation (LAMA) approach [17]. As a novel method for eccDNA synthesis, CRISPR-C is highly efficient but difficult to ensure stability [18]. Here, we synthesized eccDNA by LAMA to preliminarily verify the function of eccDNAs in different gene regions. Therefore, this paper helps bridge the gap in the existing literature and describes the chromosomal distribution and functional characterization of eccDNA in colorectal cancer.

## Results

### EccDNAs had aggregation and smaller length in CRC tissues

**Fig. 1:**
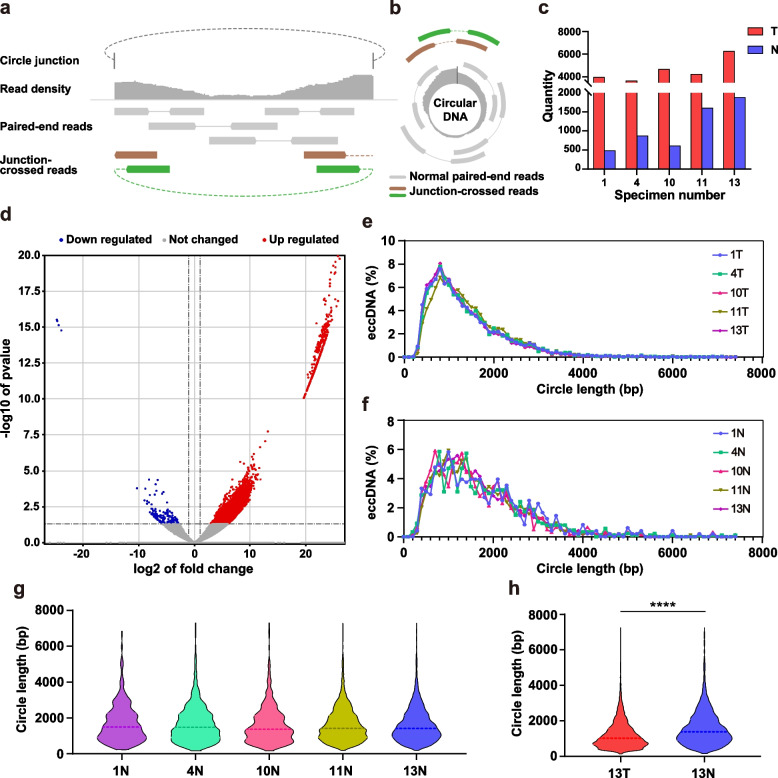
Length distribution of eccDNAs in CRC and normal tissue samples. **a** Schematic representation of eccDNA fragmentation mapping. **b** Schematic representation of circularization of tiled eccDNA. **c** Histogram of the quantity of eccDNA in each tumor group and normal group. Y-axis does not omit the intervals, only the percentages of 0–500 is increased to visualize the data in normal group. **d** Volcano plot for the comparison between the eccDNAs in tumor group and normal group. The cutoff values fold change > 2 and *P* value < 0.05 were utilized to identify differentially expressed genes. **e**-**f** Line chart of the percentage of length distribution of eccDNA in each tumor group and N. **g** Violin plots of the length distribution of eccDNA in 13 T and 13 N (*n* = 6058 in 13 T, *n* = 294 in 13 N, *P* < 0.0001). **h** Violin plots of the length distribution of eccDNA in each normal group. The dashed line is the median of each group

We used Circle-seq to assess the gene expression levels of tumor (T) and tumor-adjacent (N) tissues from 5 pairs (named 1, 4, 10, 11, 13) of CRC and tumor-adjacent tissue samples (Fig. 1a-b) [13]. When the quantity of eccDNAs in each pair of samples were compared (Fig. 1c), it was evident that the number of eccDNAs in the tumor groups was much larger than that in the tumor-adjacent tissue groups, and the majority of eccDNAs in the tumor group were highly expressed. We detected a total of 55,702 eccDNAs screening results (Fig. 1d), of which 6333 eccDNAs were differentially expressed in tumors. However, only 3594 genes were found to be present in these 6333 eccDNAs, demonstrating that multiple observed eccDNA sequences may have originated from different sites within the same gene. The length distribution of eccDNAs was then examined between the tumor and tumor-adjacent tissue samples (Fig. 1e-f). The findings revealed that the distribution consistency of eccDNA length amongst the five groups of tumor tissues was better than that of tumor-adjacent tissues. We also found that more than 95% of the eccDNAs were between 300 and 3000 bp in length, with average values in the tumor group ranging from 1286 to 1482 bp. Most tumor group eccDNAs peaked around 700–800 bp, with some having an additional peak at 400–500 bp (1 T and 4 T). In the tumor-adjacent tissue groups, eccDNA with a length of 300-3000 bp accounted for only 83% of the total, with an average length of 1712-2216 bp and disordered peaks.

The multi-peaked distribution was regularly observed in each tumor-adjacent tissue group (Fig. 1g), and the difference was more obvious when focused on 13 T and 13 N (Fig. 1h), with which a better reads mapping rate (Supplementary Fig. 1a). In comparison to tumor-adjacent samples, eccDNA in CRC tissues appeared to have a smaller, more uniform, and dense length distribution (Supplementary Fig. 1b-f).

### Highly expressed eccDNAs were convergent and contained more oncogenes

**Fig. 2:**
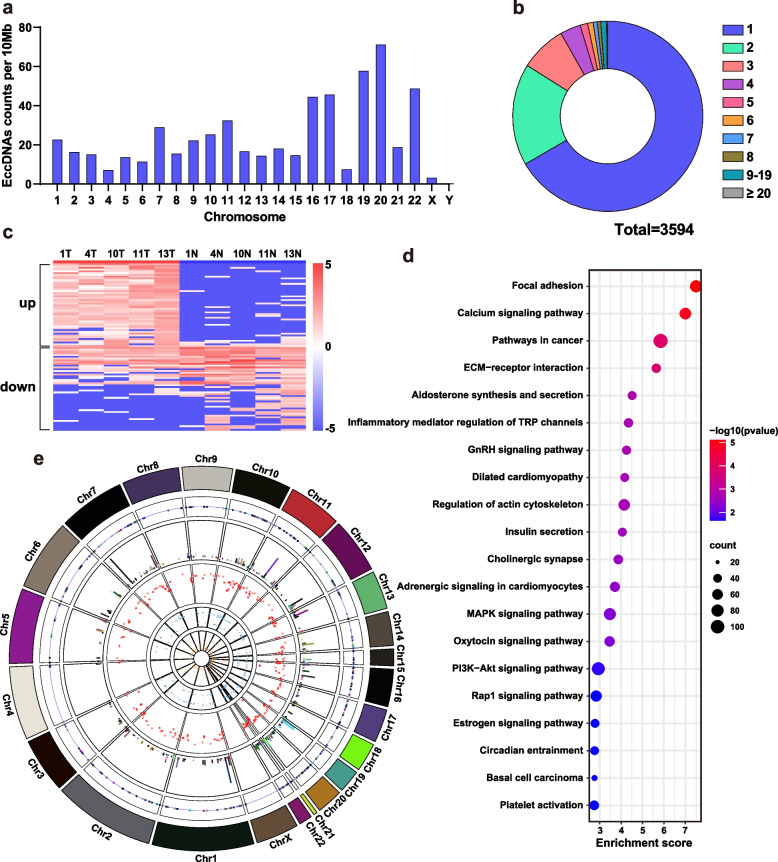
Counts distribution, gene expression and chromosomal distribution of eccDNAs in the samples. **a** Histogram of the eccDNA frequency per 10 Mb in each chromosome. **b** Pie chart of the percentages of genes (*n* = 3594) shedding different amounts of eccDNAs. **c** Heatmap consisting of the logarithm of the normalized values of the highly and lowly expressed genes in each tumor group and N (*n* = 47 in high and low expression respectively). **d** Scatter plot of enriched KEGG pathways statistics for genes (n = 3594) contained in eccDNAs in tumor group. **e** Circos plot of chromosomal distribution and expression levels of high-expressed eccDNAs in tumor group. Concentric circles are, in order from outside to inside: human chromosomes (chr1-chrX), after which the x-axis of all circles is chromosomal coordinate; chromosomal localization of eccDNAs; the basemean of the genes, with the y-axis showing the expression level; Rainfall plot of the degree of gathering of eccDNAs, and the y-axis corresponds to the minimal distance of the region to its two neighboring regions; the foldchange of the genes; the P value of the genes

In addition to the length distribution, we analyzed the counts distribution of eccDNAs in different chromosomes (Fig. 2a). There was a significant difference in the eccDNAs frequency per 10 Mb in different chromosomes. EccDNAs were shed more from chromosomes 19 and 20; but less on sex chromosomes, which was similar to the distribution previously observed in esophageal squamous cell carcinoma [19]. Since a single gene could generate different amounts of eccDNA, we calculated the counts of eccDNAs generated by different genes (Fig. 2b). The majority (66.7%) of genes producing only one eccDNA, and the number of genes producing more than one eccDNA gradually decreased with the counts of eccDNAs, consistent with previous reports [8, 19, 20].

To investigate the expression differences and chromosome distribution of genes contained in eccDNAs, 47 representative genes with high and low expression were selected respectively to evaluate their expression differences (Fig. 2c). The tumor group showed similar expression of the highly expressed oncogenes in colorectal cancer, including previously reported EGFR and ERBB2 [21–23], and the solute carrier (SLC) family. We then enriched the KEGG pathways and found that genes contained in eccDNAs were more likely to be related to the development and metastasis of tumors and classical signaling pathways (Fig. 2d).

To further analyze the chromosomal origin of eccDNA in the samples, we used Circos to enhance the visualization of Circle-seq data (Fig. 2e) [24]. The original location of eccDNA is biased toward aggregation, according to the distribution of the rainfall plot. As evidenced by the distribution of the basemean and fold change, there was no correlation between the expression level of genes contained in eccDNA and their differential degree. In the meantime, the gene expression level was higher, in regions where genes were densely distributed, although not absolutely [25].

**Fig. 3:**
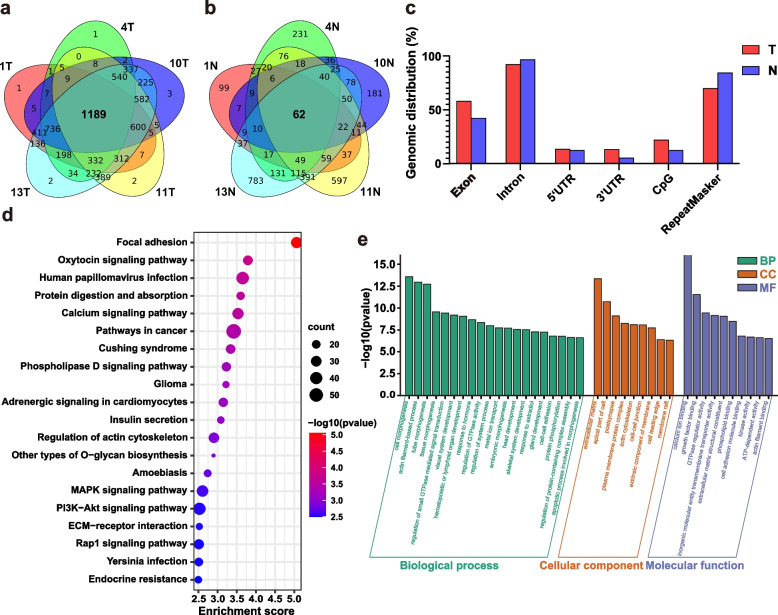
Gene regions and functional analysis of eccDNAs with concentrated high expression in CRC. **a**-**b** Venn diagram for eccDNA sequences common to each sample in tumor group and N, respectively. **c** Distribution of eccDNAs intersected in tumor group and N in the indicated genomic regions. **d** Scatter plot of enriched KEGG pathways statistics for genes (*n* = 1189) contained in eccDNAs shared in each tumor group. **e** GO functional classification of genes (n = 1189) contained in eccDNAs shared in each tumor group

We enriched the location of eccDNAs shared by 5 samples of tumor and tumor-adjacent tissue groups respectively (Fig. 3a-b) and showed the genomic regions contained in these sequences (Fig. 3c). It can be seen that, as compared to tumor-adjacent tissues, the eccDNA sequences in tumor tissues were more convergent. As compared to the genomic regions present on both tumor and tumor-adjacent tissues, the overall distribution proportion of genomic elements was similar: most sequences contained intron regions as “background”, and the majority also contained repeat regions. The presence of simple repeats was more frequently observed in the tumor-adjacent tissues, with (TG)n and (CA)n being two typical instances. Exons and CpG islands accounted for about 57.9% and 22% in the tumor tissues, respectively, compared to 42.1% and 12.3% in the tumor-adjacent tissues, indicating that eccDNA in tumor tissues tended to fall off in high-expressed genomic regions. According to the tumor group, eccDNAs shed at the 5’UTR or 3’UTR without tendency. In addition, we enriched the KEGG pathways of these genes and compared to the outcomes of the total differentially expressed genes in eccDNAs (Fig. 3d). The top 20 KEGG pathways were similar, with most of them involved in tumorigenesis, adhesion, and metastasis, as confirmed by the results of GO functional analyses (Fig. 3e).

### Partial eccDNAs affected transcriptional levels to express oncogenes

**Fig. 4:**
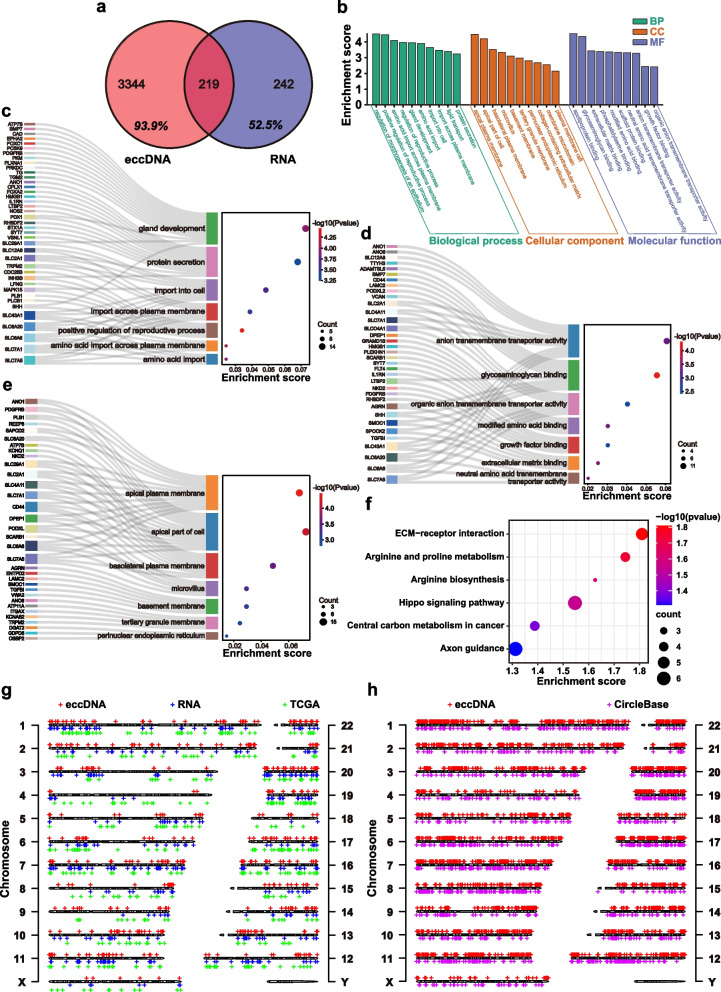
The association of genes contained in eccDNAs with RNA levels. **a** Venn diagram for genes differentially expressed in eccDNA and RNA. **b** GO functional classification of genes highly expressed in both in eccDNA and RNA. **c-e** Sankey plot for genes in biological process, cellular component and molecular function in Fig. 3b. **f** Scatter plot of enriched KEGG pathways statistics for genes (*n* = 219) highly expressed in both in eccDNA and RNA. **g** Genomic distribution of eccDNA (*n* = 500) and RNA (*n* = 461) in tumor group compared with TCGA samples (*n* = 495). **h** Genomic distribution of eccDNA in tumor group (*n* = 6275) and CircleBase (*n* = 1461)

We performed simultaneous RNA sequencing on these samples and reported that 219 genes were highly expressed in both RNA-seq (47.5%) and Circle-seq (6.1%) (Fig. 4a). Nearly half of the highly expressed genes in the transcriptional level were also differentially expressed in eccDNAs, indicating a possible link between eccDNA and RNA, which was consistent with previous research [4, 12]. We described the expression differences and 219 genes which were highly expressed in both RNA-seq and Circle-seq (Supplementary Fig. 2a). It can be seen that eccDNAs containing these genes were not highly expressed in all T groups. However, the chromosome distribution of these eccDNAs still had aggregation (Supplementary Fig. 2b).

Subsequently, we performed GO analyses on 219 genes (Fig. 4b) and identified vital biological processes, including cell proliferation, chromosome replication, and amino acid import (Fig. 4c-e). Surprisingly, we also discovered that the KEGG pathways of these 219 genes were more directed to the biological functions of arginine (Fig. 4f).

Furthermore, we compared the chromosomal location of differentially expressed eccDNAs to that of highly expressed RNAs, and we examined the transcription profile of highly expressed genes in CRC from TCGA (Fig. 4g). The chromosomal distribution of eccDNAs and RNAs was shown to be largely overlapping, indicating a similar aggregation mechanism. Due to the scarcity of reports on colorectal cancer eccDNA sequencing, we compared the chromosomal distribution of eccDNA in our samples to the gastrointestinal tumor tissues in the CircleBase (Fig. 4h), a new circular DNA database [26], and obtained comparable results to the former.

### There was a potential consistency of eccDNA in CRC tissues and cell lines

**Fig. 5:**
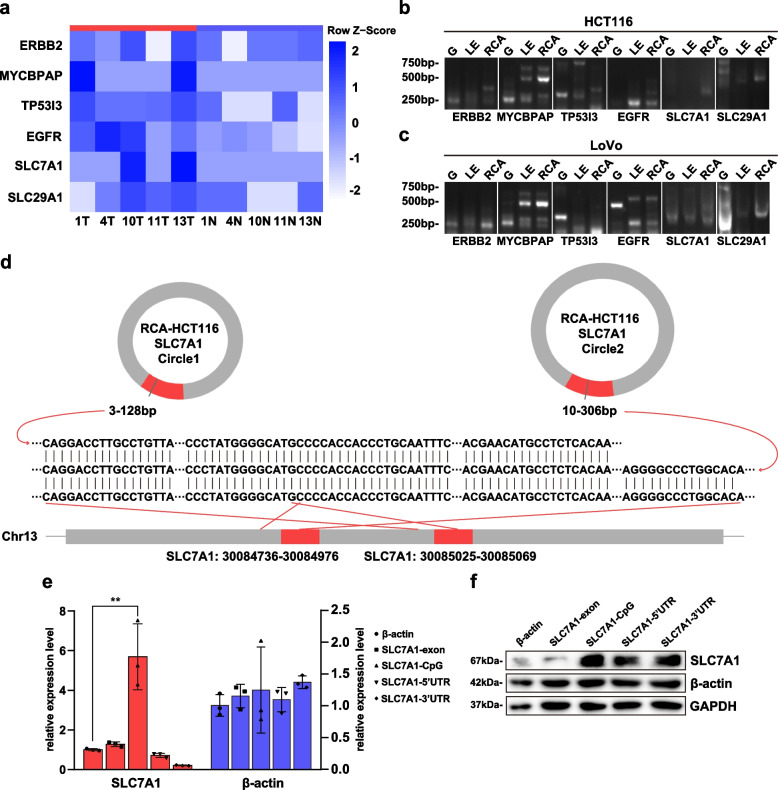
Functional identification of eccDNAs in colorectal cancer cell lines. **a** Heatmap of differential expression of genes REBB2, MYCBPAP, TP53I3, EGFR, SLC7A1, SLC29A1 in the samples. **b**-**c** DNA electrophoresis of inverse PCR for genes in Fig. 5a to verify eccDNA in HCT116 and Lovo respectively. G: genome DNA. LE: total DNA with linear DNA elimination. RCA: total DNA with linear DNA elimination after RCA. **d** Schematic representation of the sequencing results of PCR products of gene SLC7A1 in samples after RCA with matching the sequence in chr13. **e**-**f** Relative mRNA expression and protein levels of SLC7A1 and β-actin in HCT116 transfected synthetic eccDNAs

To demonstrate the existence of eccDNA in colorectal cancer cell lines (HCT116 and LoVo), we spliced the samples’ eccDNA segments of genes ERBB2, MYCBPAP, TP53I3, EGFR, SLC7A1, and SLC29A1 using IGV 2.8.10 to reveal the complete circular DNA sequences and verified their differential expression by DESeq2 (Fig. 5a). We then designed inverse PCR primers to fit the circular DNA in the samples. The primer sequences can be found in Supplementary Table 1. We also employed the primers of human β-globin gene HBB for PCR reactions to confirm linear DNA elimination by exonuclease (Supplementary Fig. 3a). The PCR results in the agarose gel electrophoresis revealed that certain bands enhanced visualization following the implementation of rolling circle amplification (RCA) using φ29 DNA polymerase (Fig. 5b-c). We sequenced all of the bands acquired from the PCR reactions and compared them to human Genomic + transcript databases to prove the target genes. Only five of the total 36 bands were matched fitting genes; two were detected off-targets to different genes, and the rest were unable to be matched to databases.

In terms of results, the two bands of HCT116 successfully matched the junction site of circular DNA (Fig. 5d), and the interrupted sequences were confirmed by human genomic databases, validating the existence of eccDNA in the CRC cell lines and its potential preference between CRC tissues and cell lines. In another case, the PCR products of both cell lines matched the gene TP53I3 at roughly 250 bp after RCA, but the junction site was missing (Supplementary Fig. 3b).

### EccDNA from CpG islands affected gene expression

We then selected SLC7A1 and artificially synthesized eccDNAs from different genomic regions, including exon 13 (sequences from Circle-seq), CpG islands, 5’UTR and 3’UTR, contrasted with the synthetic eccDNA of β-actin (sequences from Circle-seq). The synthetic sequences can be found in Supplementary Table 2. We verified the transfection effectiveness of synthetic eccDNAs in HCT116 (Supplementary Fig. 3c), and detected the transcriptional and translational levels of SLC7A1 and β-actin in this CRC cell line after transfection (Fig. 5f-g). Compared with the exon, the eccDNA of CpG islands had a significant role in promoting transcription and protein expression. The 5’UTR and 3’UTR eccDNAs may affect protein translation through extra-transcriptional pathways, as the mRNA levels were inconspicuously changed. The functional differences of eccDNAs from different genomic regions should be taken into account in further studies.

## Discussion

This study demonstrated the distribution profile and functional characterization of eccDNAs in colorectal cancer, and briefly validated in CRC cell lines. Altogether, our findings elucidated eccDNAs had aggregation and smaller length in CRC tissues, and most of them contained oncogenes, which partially affected differential expression by regulating the transcriptional level. Moreover, eccDNAs produced from different regions of the same gene affected gene expression discrepantly, especially high from CpG islands.

Comparable characteristics of length distribution in CRC tissues were reported in esophageal carcinoma and hypopharyngeal carcinoma [19, 20], with similar size and peaks. More than half of eccDNAs were less than 1000 bp, which may be presumably by-products of genetic recombination [27], indicating the genomic instability in the process of tumor development [28]. We hypothesized that it was related to the highly unstable genome in tumor tissues, whose gene recombination was purposeful and oriented, resulting in a shorter length of eccDNA and a high concentration in a specific length region. The differentiation between tumor and tumor-adjacent tissues was also demonstrated in lung cancers [1], which may suggest a novel clinical tumor early surveillance technique in the future. Although this differentiation was observed in our samples, its generalizability and will scientific validity as an emerging clinical diagnostic technique will require further sample and data validation due to the limited sample size in this study.

Apart from their length distribution, the genetic makeup of eccDNAs in CRC tissues was more convergent, which further confirmed the previous speculation that the formation of eccDNA in tumor tissues had tropism. According to KEGG pathway analysis and GO analysis, oncogenes contained in eccDNAs were intensively expressed, which were related to the pathways of tumorigenesis, development and metastasis. Therefore, we speculated that eccDNAs may drive tumor evolution and genetic heterogeneity in tumor tissues by regulating oncogene amplification in colorectal cancer.

We analyzed the genomic region distribution of eccDNAs in CRC tissues, and verified the functional effectivity of eccDNAs from different regions in vitro. EccDNA from CpG islands can up-regulate the transcriptional and translational levels of SLC7A1, but only accounts for 22% of the total. This signified that most eccDNAs are not able to manipulate gene expression by directly regulating the RNA levels [2]. However, the distribution of eccDNA genomic elements varied in different tumors, with a similar pattern observed in ovarian cancer but a greater concentration of 5’UTR and 3’UTR in esophageal cancer [1, 19].

Combined with RNA sequencing and Circle-seq, we found that partial genes were highly expressed in both eccDNA and RNA, and were enriched in arginine-related pathways. While the relationship between arginine and cancer has been widely reported [29], its association with eccDNA remains unclear and warrants further investigation. Comparing the chromosomal positions of differentially expressed genes in the eccDNA and RNA levels, we hypothesized that a small part of eccDNAs may be able to assist and promote tumorigenesis and chromosomal replication through genetic transcription and translation. Although a previous study has shown that there is no general correlation between eccDNA expression level and the transcriptional level of relative genes [12], we still propose the possibility in a small number of genes, especially oncogenes.

**Fig. 6:**
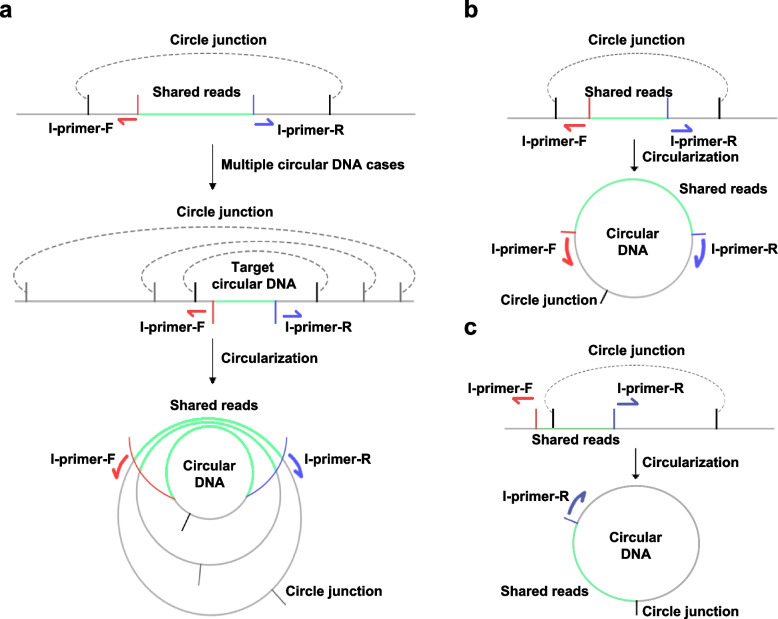
The principle and deficiency of inverse PCR for verifying eccDNAs. **a** Schematic representation of inverse PCR being off-target. **b-c** Schematic representation of inverse PCR failing to detect junction sites

We verified our discovery using inverse PCR in CRC cell lines. Admittedly, there are certain limitations in the design of primers due to inadequate specificity and the risk of being off-target [19]. The shared reads are the sequence between the two inverse primers. Theoretically, the circular DNA may be used as substrates for PCR reaction as long as it contained the shared reads (Fig. 6a), and the subsequent gel electrophoresis should result in multiple bands of varying sizes. Inverse primers may fail to correctly match randomly shed eccDNA in CRC cell lines, causing undetectable junction sites (Fig. 6b-c).

Our study has a number of limitations. First of all, the lack of sample size limited the universality of result analysis, and large-scale sequencing is needed to verify the generalization. Secondly, the association of chromosomal position between eccDNA and RNA needs to be further proved, lacking direct evidence to reinforce these results. Finally, the accuracy of inverse PCR method needs to be improved, focusing on accurately locating the junction sites of circular DNA and reducing off-target.

In conclusion, our work corroborated the amplification of eccDNA in colorectal cancer tissues at the genetic level, as well as the different distribution of eccDNAs in tumor and tumor-adjacent tissues. The single-peaked type of length distribution of eccDNAs in cancer introduced an innovative approach to clinical tumor assessment. In addition, the chromosomal origin of eccDNA was consistent with the chromosomal distribution of high-expressed genetic transcription profiles of colorectal cancer. Subsequently, we demonstrated the existence of eccDNAs in colorectal cancer cell lines and the effect distinction of eccDNAs from different genomic regions in vitro. Future research is warranted to further investigate the functional role and molecular mechanisms of eccDNA on tumorigenesis and progression.

## Materials and methods

### Clinical specimens

Our CRC tissue samples from surgical specimens were obtained from Sir Run Run Shaw Hospital, College of Medicine, Zhejiang University. 14 pairs of samples were collected and screened by our criteria. Patients in this study were selected with age over 65 years, male or female, BMI within 18 to 24, without other medical history of chronic disease and tumor. We chose CRC tissues located in right hemicolon, with pathological type belonging to moderately or poorly differentiated adenocarcinoma and IHC showing MLH1 (+), MSH2 (+), MSH6 (+), PMS2 (+), whether metastatic or not. Then we selected 5 pairs of them for this study. Tumor-adjacent tissues were distant from cancer.

### Cell culture

Human CRC cell lines (HCT116 and LoVo) were purchased from ATCC. HCT116 cells were cultured in McCoy’s 5A medium (Genom), and LoVo cells were cultured in F-12 K medium (Genom) supplemented with 10% fetal bovine serum (FBS) (Sijiqing). Cells were kept at 37 °C in a 5% (vol/vol) CO2-containing atmosphere. All cell lines were routinely tested and had negative results for mycoplasma or other pathogen contamination.

### Circular DNA preparation

We extracted genomic DNA from specimens (tissues < 25 mg or about 1 × 10^7^ cells) using MagAttract High Molecular Weight (HMW) DNA Kit (QIAGEN). Note that the specimens after added 180 ul Buffer ATL and 20ul proteinase K must be incubated at 56 °C while shaking at 900 rpm over 2 hours until completely dissolved. Then 4 ul RNase A (100 mg/mL) was added to the samples for 2 min at room temperature. After adding 150 ul Buffer AL and 280 ul Buffer MB, 40 ul fully resuspended MagAttract Suspension G was added to the mix while shaking at 1400 rpm for 2 min. The samples were then placed in a magnetic rack waiting approximately 1 min, and the genomic DNA was absorbed on the magnetic bead pellets. Then wash the magnetic bead pellets twice using 700 ul Buffer MW1 and 700 ul Buffer PM. Subsequently, 50 ul DEPC water was added to elute the DNA from the magnetic bead pellets, and then measure the concentration of the DNA.

We then used Plasmid-Safe exonuclease (NEB) to eliminate linear chromosomal DNA. 5 μg genomic DNA was mixed with 2 ul Plasmid-Safe ATP-Dependent DNase (20 units), 4 ul ATP (25 mM) and 10 ul Plasmid-Safe 10× Reaction Buffer, then added DEPC water to a total volume of 100 μL, incubating at 37 °C for 24 hours. After each 24-hour incubation, add 2 ul Plasmid-Safe ATP-Dependent DNase, 4 ul ATP and 0.6 ul Plasmid-Safe 10× Reaction Buffer into the reaction for 5 days, then heat inactivate the exonuclease by incubating at 70 °C for 30 min. Subsequently, primers of gene HBB: 5′-TATTGGTCTCCTTAAACCTGTCTTG − 3′ and 5′-CTGACACAACTGTGTTCACTAGC-3′ were used to verify linear DNA elimination by PCR reaction.

### Rolling circle amplification of circular DNA

We used Qiagen REPLI-g Mini Kit (QIAGEN) containing φ29 DNA polymerase. 5 ul fresh Buffer D1 was added to the 5 ul circular DNA sample above, incubated at room temperature for 3 min. 10 ul fresh Buffer N1 was then added to the mix to stop the reaction. Then put 30 ul prepared REPLI-g DNA Polymerase master mix (29 ul REPLI-g Mini Reaction Buffer and 1 μL REPLI-g Mini DNA Polymerase) into the sample, and incubated the reaction at 30 °C for 16 hours. After heat inactivate the reaction at 65 °C for 3 minutes, the quantity of circular DNA obtained was measured by a Qubit Fluorometer.

### Library preparation and eccDNA sequencing

The phi29-amplified DNA products were sheared with a focused ultrasonicator (Bioruptor™ Diagenode) to a mean fragment size of 200–500 base pairs; followed by purification using MolPure® Gel Extraction Kit (YEASEN). The fragmented DNA samples were used for sequencing library construction (VAHTS Universal DNA Library Prep Kit for Illumina V3, VAZYME). The library was purified by purification beads (VAZYME) and the size distribution of fragments were analyzed on Agilent 2100 Bioanalyzer (Agilent).

Next generation sequencing was performed on Illumina NovaSeq 6000 using the constructed libraries. All libraries were sequenced as 2 × 150-bp paired-end read. Raw reads were trimmed by the Trim Galore software (version 0.5.0). The cleaned reads were aligned to hg19 genome using BWA (version 0.7.17). Next the software SAMtools (version 1.8) was used to convert the aligned SAM files into BAM files. Then Circle-Map (version 1.1.3) was used to identify the circular DNA, and the finally generated BED file contained the genomic coordinate information of circular DNA. Finally, the annotation of genome position was performed using the software BEDTools (version 2.17.0).

### Synthetic eccDNAs preparation and transfection

Synthetic eccDNA sequences were identified by UCSC Genome Browser database. The linearized dsDNAs were acquired through PCR reaction and a PCR Purification Kit. The primer sequences for PCR of each genomic regions in SLC7A1 are listed in Supplementary Table 3. Synthetic eccDNAs were prepared by circularizing the dsDNAs using HiFi Taq DNA ligase (NEB), and performed in thermocyclers with the following cycles: 95 °C for 3 min, 60 °C for 10 min and 37 °C for 5 min for at least 15 cycles. The synthetic eccDNAs were digested with Plasmid-Safe ATP-dependent DNase (Lucigen) and amplified by φ29 DNA polymerase (QIAGEN) before being regained with a PCR Purification Kit. Each junction site of the synthetic eccDNAs was verified through sequencing the PCR products by inverse primers. The inverse primer sequences for PCR of each genomic regions in SLC7A1 are listed in Supplementary Table 4.

Synthetic eccDNAs transfection was carried out using PolyJet™ DNA Transfection Reagent (SignaGen, SL100688) according to the manufacturer’s instructions.

### RNA isolation, RT-qPCR and RNA-seq

Total RNAs were extracted from CRC cell lines using SteadyPure Quick RNA Extraction Kit (Accurate Biology), and cDNAs were reversed by ABScript III RT Master Mix for qPCR with gDNA Remover (Abclonal) according to the manufacturer’s instructions. The primer sequences for RT-qPCR of each gene are listed in Supplementary Table 5. RT-qPCR was conducted in triplicate with 2X Universal SYBR Green Fast qPCR Mix (Abclonal). RNA-seq libraries were obtained by the NEBNext Ultra Directional RNA Library Prep Kit (NEB) according to the manufacturer’s instructions for Illumina sequencing.

### PCR and agarose gel electrophoresis

The 1.1X T3 Super PCR Mix (Tsingke) was used to perform PCR reaction according to the manufacturer’s instructions. PCR products of gDNA, total DNA with linear elimination before and after RCA were loaded onto an agarose gel with equal volume and was conducted with vertical agarose gel electrophoresis. The results were visualized by staining with GreenView DNA Gel Stain (GeneCopoeia; 1: 10,000).

### Western blot assay

The total proteins were extracted using Cell lysis buffer for Western and IP (Beyotime) with 100x Protease inhibitor cocktail (Beyotime). Western blot was conducted as described [30]. The following primary antibodies were used: SLC7A1 (Abclonal, A14784), β-actin (Elabscience, E-AB-20031) and GAPDH (Proteintech, 10,494–1-AP).

### Circle-seq data analyses

Circle-seq used Illumina MiSeq Technology for data analyses. Briefly, the quality of the original data was evaluated using Fastqc software, then the original data were compared to the reference genome by BWA software. Samtools was used to process the sam file to fit the format required by Circle-MAP that was applied to detect eccDNA. After gene annotation to eccDNA, differential eccDNA analysis and annotated gene function enrichment analyses were performed. All the gene positions are based on GRCh37.p13 from the National Center for Biotechnology Information (NCBI). All the GO functional analyses and enriched KEGG pathways statistics were performed on DAVID Bioinformatics Resources (DAVID Functional Annotation Bioinformatics Microarray Analysis (ncifcrf.gov )).

### Statistical analyses

Statistical analyses were performed by GraphPad Prism 6. Comparisons between tumor group and N were used Student’s t test. Correlation comparisons within each group were used Pearson correlation test. All the tests were at 95% confidence intervals with *P* value setting to 0.05. Data were presented as mean ± standard error of the mean.

## Supplementary Information

**Additional file 1.**
