# Supplementary material for: Distribution and characterization of extrachromosomal circular DNA in colorectal cancer
Source: Mol Biomed. 2022 Dec 2;3:38. doi: 10.1186/s43556-022-00104-0 (PMC9718908; doi:10.1186/s43556-022-00104-0)
Supplement: Supplementary file 1 — Additional file 1. [file 43556_2022_104_MOESM1_ESM.pdf]

Supplementary information

Distribution and characterization of extrachromosomal circular DNA in colorectal cancer

Zhehang Chen[1,3,#], Yadong Qi[1,3,#], Jiamin He[1,3,#], Chaochao Xu[1,3], Qiwei Ge[1,2], Wei Zhuo[3,4,5], Jianmin Si[1,3,4,*], Shujie Chen[1,3,4,*]

Supplementary Fig. 1-3

Supplementary Fig. 1 Length distribution of eccDNA in colorectal cancer and normal tissues. a Histogram of the reads mapping rate of eccDNA in each tumor group and normal group. **b-f** Cumulative frequency plots of eccDNA in tumor group and normal group.

Supplementary Fig. 2 Chromosomal distribution and origin of genes highly-expressed both in Circle-seq and RNA-seq. a Heatmap of 219 genes highly-expressed both in Circle-seq and RNA-seq in each group. **b** Genomic distribution of 219 genes highly-expressed both in Circle-seq and RNA-seq.

Supplementary Fig. 3 Verification of eccDNA in colorectal cancer cell lines. a Gel electrophoresis result of the total DNA. HG: genome DNA of HCT116. HLE: total DNA with linear DNA elimination of HCT116. LG: genome DNA of LoVo. LLE: total DNA with linear DNA elimination of LoVo. **b** Schematic representation of the sequencing results of the PCR products of gene TP53I3 in samples after RCA with matching the sequence in chr2. **c** DNA electrophoresis of inverse PCR for junction sites of synthetic eccDNAs to prove transfection effectivity in HCT116. G: genome DNA. LE: total DNA with linear DNA elimination. RCA: total DNA with linear DNA elimination after RCA.

Supplementary Fig. 1

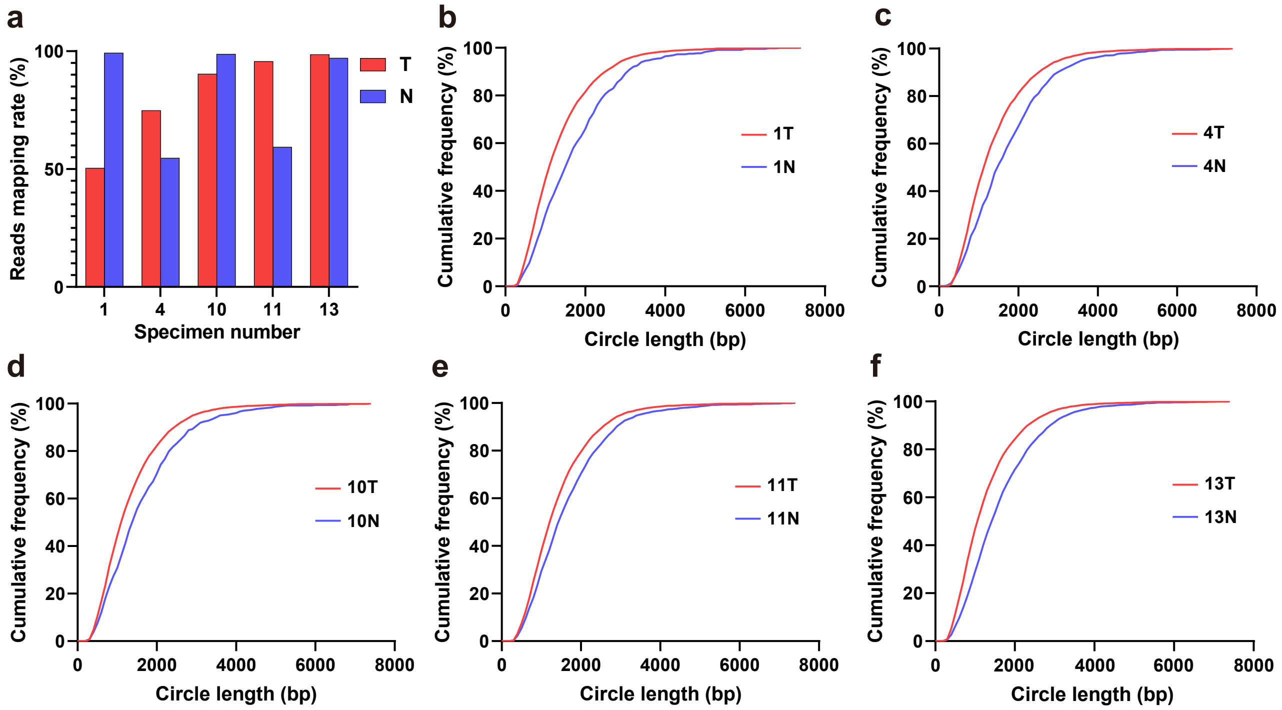

Supplementary Fig. 2

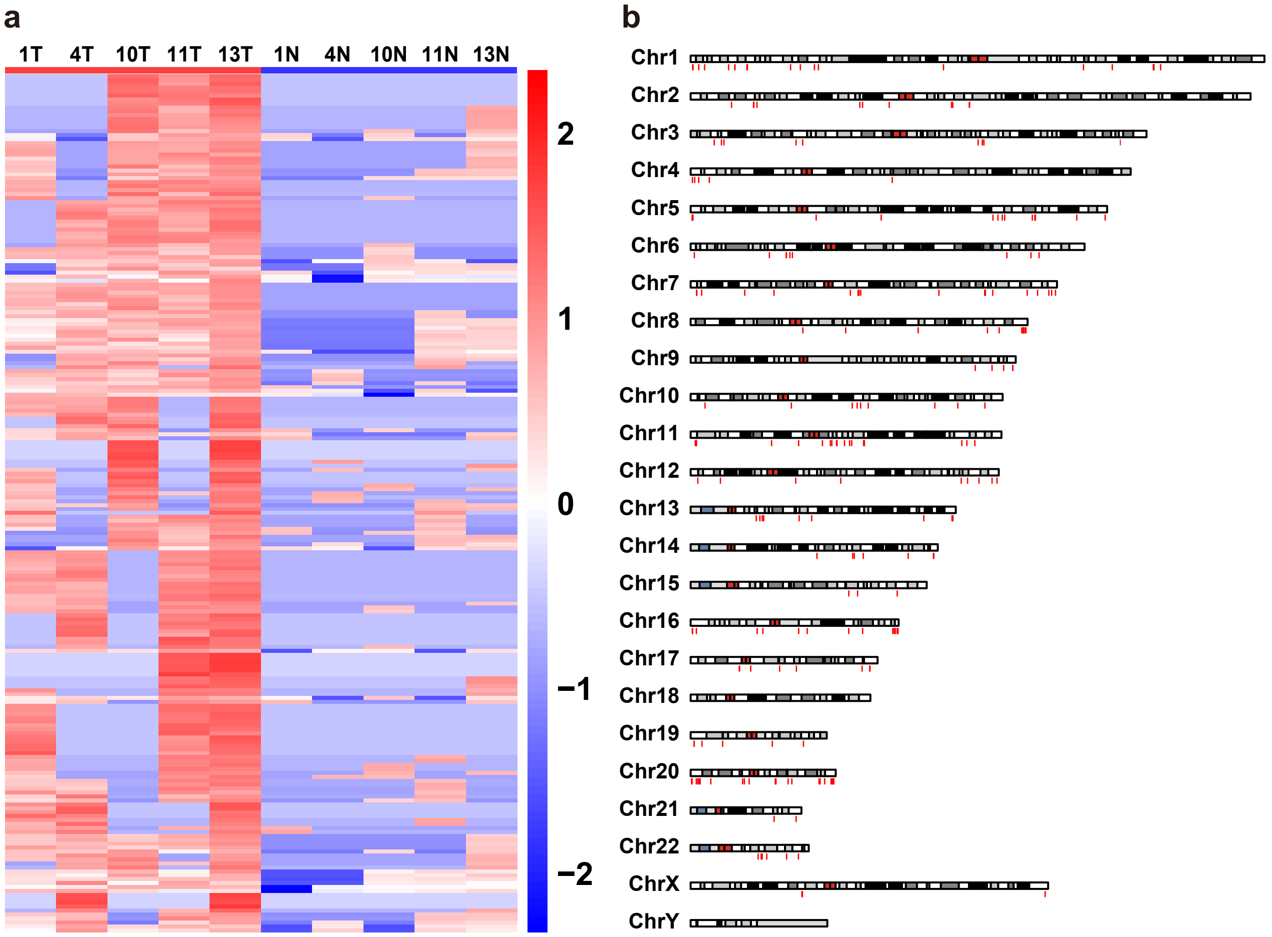

Supplementary Fig. 3

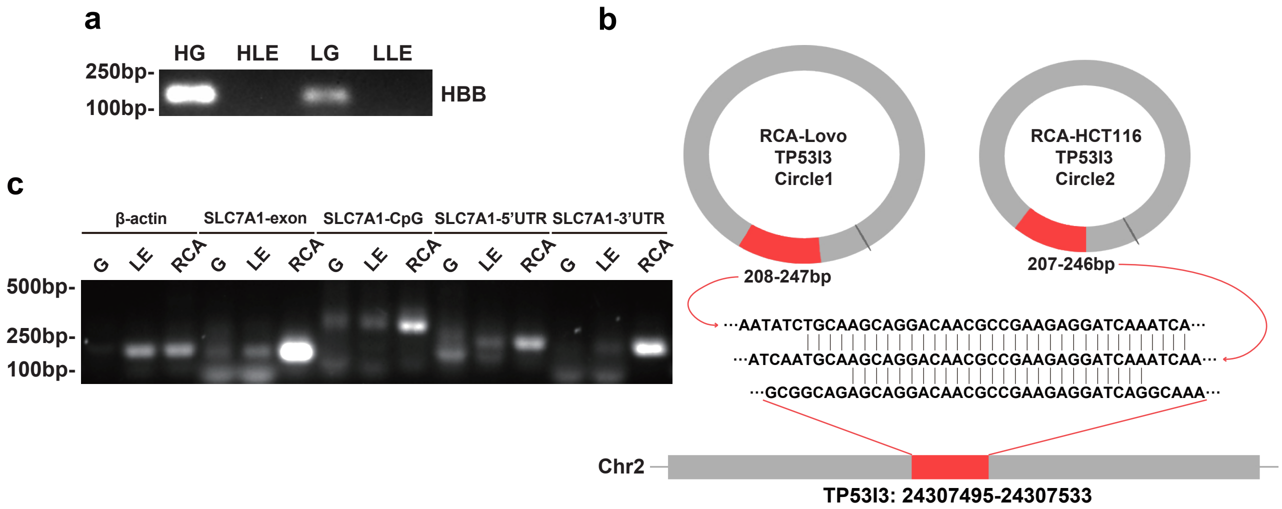

Supplementary table 1-5

Supplementary Table 1

Sequences of inverse primers[1] and PCR reaction[2]

<table>
<tr><th>Gene</th><th>Primer</th><th>Sequences</th><th>PCR conditions</th></tr>
<tr><td rowspan="2">ERBB2</td><td>FRD:</td><td>5’-GCCGTGGCTCACACCTGTAATC-3’</td><td rowspan="12">1. 98℃ for 2 min
2. 35 cycles of:
98℃ for 10 sec
60℃ for 15 sec
72℃ for 10 sec
3. 72℃ for 5 min
4. 4℃ for save</td></tr>
<tr><td>REV:</td><td>5’-AGCAAGTTCCCAGACCTCCAGG-3’</td></tr>
<tr><td rowspan="2">MYCBPAP</td><td>FRD:</td><td>5’-TTCGGCAGAGTGGCGGCAAT-3’</td></tr>
<tr><td>REV:</td><td>5’-AGAGCAAACAACCCAGCAGAGC-3’</td></tr>
<tr><td rowspan="2">TP53I3</td><td>FRD:</td><td>5’-AACCTCTTGGCGGGCGGATT-3’</td></tr>
<tr><td>REV:</td><td>5’-TGATCCTCTTCGGCGTTGTCCT-3’</td></tr>
<tr><td rowspan="2">EGFR</td><td>FRD:</td><td>5’-ACCTCCATCAGTGGCGATCTCC-3’</td></tr>
<tr><td>REV:</td><td>5’-GCCGCAATGTGGACAATACAGG-3’</td></tr>
<tr><td rowspan="2">SLC7A1</td><td>FRD:</td><td>5’-GGCAGGCTTGCTTTAGCATTGA-3’</td></tr>
<tr><td>REV:</td><td>5’-TGTGAGAGGCATGTTCGTGTCT-3’</td></tr>
<tr><td rowspan="2">SLC29A1</td><td>FRD:</td><td>5’-CCTGCCCTGGGTGGTATGAACT-3’</td></tr>
<tr><td>REV:</td><td>5’-GCTAAAGGCTTGGTTCGGAGGC-3’</td></tr>
</table>

[1] Oligonucleotide primers were designed by Primer Premier 6.0

[2] PCR reaction was performed on ProFlex™ PCR System (Thermo Fisher)

Supplementary Table 2

Sequences of the synthetic eccDNAs

eccβ-actin: 358bp

GCTCATTGCCAATGGTGATGACCTGGCCGTCAGGCAGCTCGTAGCTCTTCTCCAGGG
AGGAGCTGGAAGCAGCCGTGGCCATCTCTTGCTCGAAGTCCAGGGCGACGTAGCAC
AGCTTCTCCTTAATGTCACGCACGATTTCCCGCTCGGCCGTGGTGGTGAAGCTGTAG
CCGCGCTCGGTGAGGATCTTCATGAGGTAGTCAGTCAGGTCCCGGCCAGCCAGGTC
CAGACGCAGGATGGCATGGGGGAGGGCATACCCCTCGTAGATGGGCACAGTGTGGG
TGACCCCGTCACCGGAGTCCATCACGATGCCAGTGGTACGGCCAGAGGCGTACAGG
GATAGCACAGCCTGGATAGC

eccSLC7A1-exon: 335bp

CCCCACCACCCTGCAATTTCCCCGTCCTCGGAACTGTCTCACGAGACCACCAGCGC
ACAGACACGAACATGCCTCTCACACACCCCAGAACGCAGCTGTGCTGGAGTTGGGC
ACAAAGGCCCCTCACTCCCTGTCTTCATGGTGTGGGTTCTTGGCTGCCAACAGAACT
GTCAGCCTTTATTAAGAAGGAAGGGTCTGTGTAAAAATGGGATGTGTGAATCAGAA
GAGGGGCCCTGGCACAGAGCCAGGCAGGCTTGCTTTAGCATTGAGTCCTGCGTCGC
GCAATGCCCAGGACCTTGCCTGTTAAGTCTGGGTGGGGCCCCTATGGGGCATGT

eccSLC7A1-CpG: 382bp

CCCGGAGAACCGGCTGCAGAGCCGAGGGTGGGCTGGGGCTGCAGGTCAAGTAATT
GCACCTTTGGCTGCAGTGAGGGTGTGGACGCAGGTGGTTTCTGTTGCACTGGTGGG
GAGGGTGGGGTGCCTCCCGGTCCTCTGGGGGCGTCCCTCGGGGCTGCTGCCACCTC
CGGGGGGCGGGGCTGTGCGTCACTTGCACTGGTCCAAGTTGCCGTCAGGAGTCCTT
GCTTGGTCGGCATCCAGGGACGCCTCCTCGCTGTGCCACAGGCCATAGCCAAAGTA
GATGATGAAGCCTGCGGGCCGACAGCAGAGACGGGCGTGAACAGACCGCCGGTTG
CACCACCGCAGGCAGGGCTCAGAGCCCAGGGCGGTCCCACAGCAAACC

eccSLC7A1-5'UTR: 387bp

GCACTGCTGATGAAACCTGGCGCCGGAACCCGCCAGCCCTCGGCGCCCATTCAGTC
CGCGCAGGCAGGTGTGAGCAGCGGGTCAACTACCTGGCAGGCGCGCACGCGGCCG
CGGGCTCCCGCTAACCGCAGCCTCCACTCCTCTCCCCGCGCGCCGCGCCCCCGCCCC
GCCCCGCCCCGCCCGGTCTCGCCGGCCGAGCGTCCGTTGGTCCTTGAGCGCGTCCG
ACAGTCTGTCTGTTCGCGATCCTGCCGGAGCCCCGCCGCCGCCGGCTTGGATTCTGA
AACCTTCCTTGTATCCCTCCTGAGACATCTTTGCTGCAAGATCGAGGCTGTCCTCTGG
TGAGAAGGTGGTGAGGCTTCCCGTCATATTCCAGCTCTGAACAGCAAC

eccSLC7A1-3'UTR: 385bp

TTCTTCTACTGAACATGGAGCCATTATTAAGAGTTGTGTGTTTTTATTATGTACATTT
GTATATTTTTTTGCTTGTTTGATGTTCTATTTTTCTAATAGTTTTCTTTTAGTTTCTTAAA
GTTGTGATACTAGATTTAGATTCTGATGCTAACTGCAAATCAGGTTGGTCTCTGCTGG
GTCTCTCCTGCTTTTATTTTACTTTAAGGACAAGTGTAGTTGTCGTCCACCACCTTTC
AAAAAATGTGAAACTGCCCTGCCTCCCCTTTTTGCTGACAACACTGTGTACATTGAC
CACTTCCTACCATACTTTATGTTGTAAAATCAAACTCTTTTGTGGTACATTATCTCATG
CTTCTGCAAATTCGAATAAATTCTATGGCTTCC

Supplementary Table 3

Sequences of primers[1] and PCR reaction[2]

Name	Primer	Sequences	PCR conditions
eccβ-actin	FRD:	5’ CCAGAGGCGTACAGGGATAGCA 3’	1. 98℃ for 2 min<br>2. 50 cycles of:<br>98℃ for 10 sec<br>60℃ for 15 sec<br>72℃ for 10 sec<br>3. 72℃ for 5 min<br>4. 4℃ for save
	REV:	5’ GCGGGAAATCGTGCGTGACAT 3’	
eccSLC7A1-exon	FRD:	5’ CCCCACCACCCTGCAATTTCCC 3’	
	REV:	5’ TGTGAGAGGCATGTTCGTGTCT 3’	
eccSLC7A1-CpG	FRD:	5’ CACTGGTCCAAGTTGCCGTCAG 3’	
	REV:	5’ CAGAAACCACCTGCGTCCACAC 3’	
eccSLC7A1-5’UTR	FRD:	5’ TTGCTGCAAGATCGAGGCTGTC 3’	
	REV:	5’ AGAGGAGTGGAGGCTGCGGTTA 3’	
eccSLC7A1-3’UTR	FRD:	5’ CAAGTGTAGTTGTCGTCCACCA 3’	
	REV:	5’ CACAACTCTTAATAATGGCTCC 3’	

[1] Oligonucleotide primers were designed by Primer Premier 6.0

[2] PCR reaction was performed on ProFlex™ PCR System (Thermo Fisher)

Supplementary Table 4

Sequences of inverse primers[1] and PCR reaction[2]

Name	Primer	Sequences	PCR conditions
eccβ-actin	FRD:	5’ GCTCATTGCCAATGGTGATGAC 3’	1. 98℃ for 2 min<br>2. 50 cycles of:<br>98℃ for 10 sec<br>60℃ for 15 sec<br>72℃ for 10 sec<br>3. 72℃ for 5 min<br>4. 4℃ for save
	REV:	5’ GCTATCCAGGCTGTGCTATCCC 3’	
eccSLC7A1-exon	FRD:	5’ GGCAGGCTTGCTTTAGCATTGA 3’	
	REV:	5’ ACATGCCCCATAGGGGCCCCAC 3’	
eccSLC7A1-CpG	FRD:	5’ CCCGGAGAACCGGCTGCAGAGC 3’	
	REV:	5’ GGTTTGCTGTGGGACCGCCCTG 3’	
eccSLC7A1-5’UTR	FRD:	5’ GCACTGCTGATGAAACCTGGCG 3’	
	REV:	5’ GTTGCTGTTCAGAGCTGGAATA 3’	
eccSLC7A1-3’UTR	FRD:	5’ TTCTTCTACTGAACATGGAGCC 3’	
	REV:	5’ GGAAGCCATAGAATTTATTCGA 3’	

[1] Oligonucleotide primers were designed by Primer Premier 6.0

[2] PCR reaction was performed on ProFlex™ PCR System (Thermo Fisher)

Supplementary Table 5

Sequences of primers[1] and RT-qPCR reaction[2]

Gene	Primer	Sequences	RT-qPCR conditions
SLC7A1	FRD:	5'GCCTGTGCTATGGCGAGTTT3'	1. 95℃ for 3 min<br>2. 50 cycles of:<br>95℃ for 5 sec<br>60℃ for 30 sec<br>3. 95℃ for 5 sec<br>60℃ for 1 min<br>4. 50℃ for 30 sec
	REV:	5'ACGCTTGAAGTACCGATGATGTA3'	
xβ-actin	FRD:	5'TGCTGTCCCTGTATGCCTCTGG3'	
	REV:	5'GGAACCGCTCGTTGCCAATAGT3'	
GAPDH	FRD:	5'CGGATTTGGTCGTATTGGG3'	
	REV:	5'CTGGAAGATGGTGATGGGATT3'	

[1] Oligonucleotide primers were designed by Primer Premier 6.0

[2] RT-qPCR reaction was performed on ROCHE LightCycler480 System (Rotor gene 6000 Software, Sydney, Australia).
